# Effect of a 3-Weeks Training Camp on Muscle Oxygenation, $\dot{\text{V}}$O_2_ and Performance in Elite Sprint Kayakers

**DOI:** 10.3389/fspor.2020.00047

**Published:** 2020-04-28

**Authors:** Myriam Paquette, François Bieuzen, François Billaut

**Affiliations:** ^1^Département de kinésiologie, Université Laval, Québec, QC, Canada; ^2^Institut National du Sport du Québec, Montréal, QC, Canada

**Keywords:** muscle oxygenation, oxygen extraction, peripheral adaptations, near infrared spectroscopy, training load

## Abstract

**Purpose:** Peripheral adaptations, as assessed via near-infrared spectroscopy (NIRS) derived changes in muscle oxygenation (SmO_2_), are good predictors of sprint kayak performance. Therefore, the goal of the present study was to assess changes in SmO_2_ and $\dot{\text{V}}$O_2_ following a training camp in elite sprint kayakers to evaluate if the training prescribed elicits peripheral adaptations, and to assess associations between training-induced changes in physiological responses and performance.

**Methods:** Eight male elite sprint kayakers, members of the Canadian National Team, performed a 200-m and 1,000-m on-water time trial (TT) before and after a 3-weeks winter training camp. Change in performance, $\dot{\text{V}}$O_2_ and SmO_2_ of the *biceps brachii* were assessed in relation to training load.

**Results:** Training load and intensity were increased by ~20% over the course of the training camp, which resulted in a 3.7 ± 1.7% (ES 1.2) and 2.8 ± 2.4% (ES 1.3) improvement in 200-m and 1,000-m performance, respectively. Performance improvement in the 200-m was concomitant to a reduced SmO_2_, an increased $\dot{\text{V}}$O_2_ peak and an increased reoxygenation rate after the TT. The 1,000-m TT performance improvement was concurrent with a reduced SmO_2_ in the last half of the TT and an increased $\dot{\text{V}}$O_2_ in the first minute of the TT.

**Conclusion:** Our results strongly suggest that peripheral skeletal muscle adaptations occurred in these athletes with the proposed training plan. This further attests the benefit of using portable NIRS as a monitoring tool to track training-induced adaptations in muscle oxygen extraction in elite athletes.

## Introduction

In sprint kayak, individual Olympic events are 200-m and 1,000-m races (~34 s and ~210 s) for men. Using the accumulated oxygen deficit method, aerobic contribution in highly-trained to international-level canoe-kayak athletes has been estimated to be ~37% for 200-m and ~85–87% for 1,000-m events (Byrnes and Kearney, 1997; Zamparo et al., 1999; Zouhla et al., 2012). In 1,000-m events, athletes perform at 102% of power at $\dot{\text{V}}$O_2_max (Zamparo et al., 1999), and therefore, $\dot{\text{V}}$O_2_max, or maximal aerobic power, and lactate threshold have been shown to correlate strongly with this performance (Tesch, 1983; Fry and Mortin, 1991; Bishop, 2000; Michael et al., 2008). Anaerobic fitness, as measured by the work done during a 30-s and a 2-min ergometer test, has also been associated with 1,000-m performance in male kayakers ranging from club to international level (van Someren and Howatson, 2008). Performance in the 200 m has been related to upper-body dimensions (van Someren and Palmer, 2003; van Someren and Howatson, 2008), upper-body strength (van Someren and Palmer, 2003; Pickett et al., 2018), and different measures of anaerobic capacities: peak power, total work and fatigue index in a 30-s ergometer test (van Someren and Palmer, 2003; van Someren and Howatson, 2008), and total work in a 2-min ergometer test (van Someren and Palmer, 2003). Performance in the 200-m TT has also been related to maximal aerobic power and/or $\dot{\text{V}}$O_2_max in some (Borges et al., 2015; Paquette et al., 2018; Pickett et al., 2018), but not all (van Someren and Palmer, 2003) studies, suggesting aerobic fitness may also partly contribute to performance in 200-m races.

The arrival of affordable and portable near-infrared spectroscopy (NIRS) monitors has increased accessibility to muscle oxygenation measures during exercise, and a few studies have assessed changes in muscle oxygenation during kayaking exercise (Dascombe et al., 2011; Borges et al., 2015; Paquette et al., 2018). In junior kayakers, there was a moderate correlation between maximal O_2_ extraction in the *latissimus dorsi* during an incremental test on a kayak ergometer and both 200-m and 1,000-m performance (Borges et al., 2015). In a more recent study, muscle oxygenation has been assessed in *biceps brachii, latissimus dorsi* and *vastus lateralis* during 200-m, 500-m, and 1,000-m time trials (TT) in sprint canoe-kayak athletes ranging from club to international level (Paquette et al., 2018). The results from this study suggest that peripheral adaptations, as assessed via NIRS-derived changes in muscle oxygenation, may be stronger predictors of canoe-kayak performance compared to cardiac output or $\dot{\text{V}}$O_2_max in both short and long events (Paquette et al., 2018).

These studies highlight the importance of peripheral adaptations for kayak performance, particularly in the sport-specific training period. Acute and specific changes in muscle oxygenation during various high-intensity interval training sessions suggest that some of the sessions typically performed by kayakers have the potential to induce peripheral adaptations (Paquette et al., 2019). However, to our best knowledge, training-induced chronic changes in muscle oxygenation parameters have not been assessed in kayak. A few studies have investigated the changes in muscle oxygenation in cyclists (Neary et al., 2002) and team-sport athletes (Buchheit and Ufland, 2011; Jones and Cooper, 2014; Jones et al., 2015; Delextrat et al., 2018), during running or cycling exercises, following a period of high-intensity training or a taper block, and have mostly reported a positive association between changes in muscle oxygenation parameters and performance improvements. Since kayak is an upper-body dominant sport, and skeletal muscles in the arms typically display larger cross-sectional area of type II muscle fibers and lower oxidative enzyme activity (Mygind, 1995), response to training in other modalities may not be directly applicable to kayaking exercise.

Furthermore, most of the studies assessing the associations between physiological adaptations and performance in kayak have been conducted on either junior athletes (Borges et al., 2015) or athletes from a wide range of performance levels (van Someren and Palmer, 2003; van Someren and Howatson, 2008; Paquette et al., 2018; Pickett et al., 2018). As described by Pickett et al. (2018), associations between physiological parameters and performance derived from athletes coming from various levels may no longer apply when considering only elite athletes. Thus, in addition to the need to assess specific training-induced changes in kayakers, there is a need to better understand performance indicators within a group of elite athletes.

Therefore, the goal of the present study was to assess changes in physiological parameters—muscle oxygenation and $\dot{\text{V}}$O_2_—following a 3-weeks winter training camp in elite kayakers to (i) evaluate if the training prescribed to elite sprint kayakers does readily elicit peripheral adaptations, and (ii) examine associations between training load, training-induced changes in physiological parameters and change in on-water performance. It was hypothesized that intensification of training during a 3-weeks training camp would induce peripheral adaptations in elite kayakers, and that performance improvements following training would be related to changes in muscle oxygenation parameters.

## Materials and Methods

### Subjects

Eight male elite sprint kayakers participated in this study. Participants were 24 ± 5 years of age (range 18–34 years old) and weighted 84.8 ± 5.4 kg. They were all members of the Canadian National Team and had 6 ± 5 years (range 1–17 years) of experience racing at the international level. Athletes had different specialities (4 were 1,000-m specialists, 1 was 200-m specialist, and 3 were racing in the K4 500 m), but were all following the same training plan at that time of the year. This study was approved by the ethical committee of Laval University and was conducted in accordance with the principles established in the Declaration of Helsinki, with verbal and written informed consent obtained from all participants.

### Experimental Design

All testing sessions were performed on the same artificial lake with an official regatta course during a winter training camp in Florida, USA. Since kayaking is not possible during winter months in Canada, athletes perform most of their specific preparation during training camps held in southern countries. Most of the specific high-volume and high-intensity training is performed during these camps, and we can therefore hypothesize that most race-specific adaptations also occur during these camps. The athletes performed a 200-m TT and a 1,000-m TT on separate days, at the end of a recovery week, in Florida. They then completed a 3-weeks training camp in Florida, and repeated the 2 TTs at the end of the next recovery week on the same course used previously. All athletes completed the 200-m TT, while the 200-m specialist did not perform the 1,000-m TT. Hence, data is presented for 8 athletes for the 200-m TT and for 7 athletes for the 1,000-m TT. During the 3-weeks training camp, kayak sessions were either base endurance, maximal aerobic power or speed sessions. This experimental 3-weeks training camp was preceded by another 3-weeks training block in Florida where the main goal was to achieve a high volume of base endurance work on the water, which was preceded by a general training block performed at home, focusing on strength training and base endurance work (mainly performed on the kayak ergometer or in cross-country skiing).

### Methodology

#### Training Load

Training load was recorded by self-reported questionnaire during the 3-weeks training camp and during the previous 9 weeks. Every day, within 30 min of the last training session, athletes filled an online questionnaire where they had to answer three questions for every training session performed on that day: session type (kayak, strength training, or other), session duration (in min), and rate of perceived exertion (RPE, modified Borg scale) (Foster et al., 2001). For each training session, session RPE (sRPE) was calculated, as the product of session duration and RPE (Foster et al., 2001). Weekly load was then computed as the sum of sRPE for the week, for each training session type. Weekly volume was the total training duration, in hours. Average RPE was calculated for each training week and considered as an index of training intensity. Session frequency was the total number of training sessions by week, for each training type. Average weekly load, duration, intensity and session frequency for the 3-weeks training camp and for the two previous 3-weeks training blocks were calculated. Kayak ergometer sessions were considered kayak sessions, but only happened during the first 3-weeks training block.

#### 200-m and 1,000-m TT

The TTs were completed on a marked course, protected from the wind and waves. The TTs were completed on the same days for all athletes, and the times and days were chosen in order to get minimal wind during trials (<5 m/s). Water temperature and wind speed were measured before and after each race. All participants performed their habitual pre-race warm-up before each TT. Expired air was continuously recorded using a mixing chamber portable gas analyzer (K5, Cosmed, Rome, Italy). The device was calibrated according to manufacturer guidelines before every test. $\dot{\text{V}}$O_2_peak for each TT was defined as the highest values achieved over a 30-s period during the test. Blood lactate concentration was measured 1 and 3 min after the end of each TT. An additional measurement was performed 5 min after the 200-m TT only. Blood was collected on the earlobe using a portable lactometer (The Edge Handheld Lactate Analyser, Woodley Equipment Company LTD, Lancashire, United Kingdom). Asepsis was performed with 70% ethylic solution on the distal portion of the earlobe prior to collection, and puncture was performed using disposable lancets.

#### Stroke Parameters

An Inertial Measurement unit (IMU; MMS2, Motus design, Victoria, Canada) was fixed on the boat using Velcro. The raw acceleration and GPS-derived speed data was analyzed using the Motus Review software (v. 2.0.11, Motus Design, Victoria, Canada), to compute stroke-by-stroke speed, stroke rate, and stroke length.

#### Muscle Oxygenation

During all TTs, a NIRS monitor (Moxy monitor, Fortiori Design, Minnesota, USA) was used to assess changes in muscle oxygenation. The Moxy monitor employs four wave-lengths of near-infrared light (680, 720, 760, and 800 nm), with source-detector spacing of 12.5 and 25.0 mm (McManus et al., 2018). The NIRS monitor was placed on the *biceps brachii* of the athlete's dominant limb, in the middle of the muscle belly (8 to 12 cm above the elbow fold) and parallel to the muscle fiber orientation. While upper-body muscles primarily involved in the kayak stroke are the *latissimus dorsi, triceps brachii* and the anterior deltoids (Fleming et al., 2012), *biceps brachii* also contributes to the force production, as suggested by the important muscle deoxygenation that occurs during kayaking in this muscle (Paquette et al., 2018). The *biceps brachii* is an easier muscle than the *latissimus dorsi* to assess with NIRS, since the monitor placement is easier and signal quality more optimal. The NIRS monitor was attached and secured with a double-sided adhesive disk and an adhesive patch, and covered by a dark bandage to reduce the intrusion of extraneous light. The monitor position was recorded and replicated during post-training measurements. Skinfold thickness at the site of NIRS measurement was measured using a skinfold caliper (Harpenden Ltd.) to ensure that the skinfold thickness was <1/2 the distance between the emitter and the detector (25 mm). The raw muscle O_2_ saturation (SmO_2_) signal, which represents the balance between O_2_ delivery and extraction by the muscle, and total heme concentration [THb], an indicator of local blood volume (Ferrari et al., 2011), were treated using a smooth spline filter to reduce the noise created by movement (Rodriguez et al., 2018). Baseline SmO_2_ and baseline [THb] were computed as a 30-s average when subjects were seated still on the kayak before the beginning of the test. As described elsewhere (Paquette et al., 2018), minimum SmO_2_ (SmO_2_min) was the lowest 5-s average SmO_2_ reached during the TT, and maximum SmO_2_ was the highest 5-s average SmO_2_, typically reached in the minutes following TT completion. Average SmO_2_ from 30 to 120 s after the end of each TT was used to assess recovery SmO_2_. For each TT, baseline SmO_2_ and SmO_2_ min are expressed as % of maximal SmO_2_. Change in SmO_2_ (ΔSmO_2_) and change in [THb] (Δ[THb]) were calculated as the difference between SmO_2_min and baseline SmO_2_, or the difference between maximum [THb] and baseline [THb]. At the onset of each TT, a linear model was used to assess rapid changes in SmO_2_ (Bae et al., 2000). The SmO_2_ vs. time values were modeled from the start to the 12th second of exercise (Buchheit et al., 2012). The slope of the relationship was retained as an index of deoxygenation rate (deoxy rate) (Bae et al., 2000). During high-intensity exercise, muscle oxygenation decreases rapidly in the first seconds of exercise (phase I), then levels off for the last part of the effort (phase II) (Bae et al., 2000). Therefore, the phase II slope corresponded to the slope of the SmO_2_ vs. time relationship during the second phase, from the 12th second to the end of the TT. Similarly, reoxygenation rate (reoxy rate) was taken as the slope of the linear part of the SmO_2_ vs. time curve, within the first 30-s period following the end of exercise [adapted from Buchheit and Ufland (2011)].

### Statistical Analysis

Means and standard deviations were calculated for performance and physiological parameters in pre- and post-training trials. The typical percent variability (coefficient of variation) of performance for top athletes in sprint canoe-kayak over a season is 1.0% (Malcata and Hopkins, 2014). Therefore, performance improvement in the current study was compared to the smallest worthwhile change in performance, determined as 0.3 times the competition-to-competition variability score, so 0.3% in sprint kayak (Hopkins et al., 2009). Performance enhancement was also compared to the average difference between 1st and 4th place in individual sprint canoe-kayak events during the Rio Olympic Games (1.4%). The 200-m TT was further divided into two equal-length segments to analyse physiological parameters and into four 50-m segments for stroke analysis. The 1,000-m TT was divided into five equal-length segments to analyse physiological parameters and into five 200-m segments for stroke parameters. Average SmO_2_, $\dot{\text{V}}$O_2_ and stroke parameters over these segments were compared between pre- and post-training trials using paired *T*-test. Cohen's d effect sizes (ES) and 90% confidence intervals were also computed for differences in means between pre- and post-training measures and ES of 0.2, 0.6, 1.2, and 2.0 were considered small, moderate, large and very large differences, respectively. Pearson correlations were calculated to assess associations between physiological changes and performance improvements, and associations between training load and physiological changes. Correlation coefficient of >0.1, >0.3, >0.5, >0.7 and >0.9 were considered small, moderate, large, very large and nearly perfect (Hopkins et al., 2009).

## Results

### Training Load

**Figure 1:**
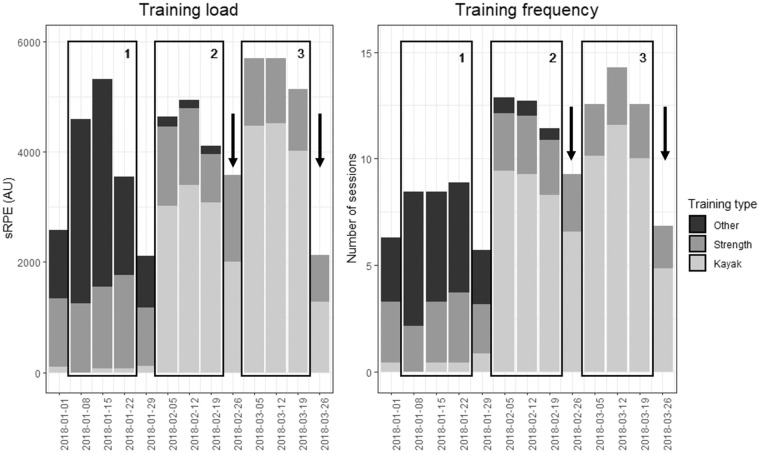
Training load and training frequency by training type during the 3-weeks training camp (block 3) and the two previous 3-weeks training blocks (blocks 1 and 2) for the period of January 2018 to March 2018. Numbered rectangles represent analyzed training blocks. Blocks 1 and 2 represent the two 3-weeks training blocks preceding the 3-weeks training camp (block 3). Each training block was preceded by a recovery week. Arrows represent the weeks where the 200-m and 1,000-m TTs were performed, before and after training block 3. sRPE, session RPE (RPE * session duration), in arbitrary units.

The training load for the 3-weeks training camp (block 3) was compared to the training load of the two previous 3-weeks training blocks (blocks 1 and 2). Figure 1 displays weekly training load and frequency in these three training blocks and Table 1 details their characteristics. During the camp, average weekly load was increased by 23% compared to the first and by 21% compared to the second training block, but these differences did not reach statistical significance (*p* = 0.08 and *p* = 0.11, respectively). Training volume did not vary across blocks, but training frequency increased in the training camp compared to both blocks, and subjective training intensity increased compared to block 2. When considering only the kayak sessions, training load, frequency and intensity were increased during the camp compared to the two previous training blocks. Strength training did not significantly vary across training blocks, and training load from other training modalities was decreased in the training camp compared to the first training block, but was similar to the second training block.

**Table 1:** Training load in the 3-weeks training camp (block 3) and the two previous 3-weeks training blocks (blocks 1 and 2).

	**Block 1**	**Block 2**	**Block 3**
Dates	Jan-8 to Jan-28	Feb-5 to Feb-25	Mar-5 to Mar-25
Load (AU/week)	4488 ± 1631.7	4562 ± 1275.9	5505.6 ± 1524.4
Frequency (sessions/week)	8.6 ± 1.8^*^	12.3 ± 2.5^*^	13.1 ± 2.8
Volume (h/week)	13.4 ± 3.8	14.8 ± 3.1	15.3 ± 3.8
Intensity (average RPE/week)	5.0 ± 0.5	4.9 ± 0.6^*^	5.9 ± 0.6
**KAYAK**			
Load (AU/week)	47.4 ± 149.8^*^	3169 ± 1194.3^*^	4336.7 ± 1351.4
Frequency (sessions/week)	0.3 ± 0.9^*^	9.0 ± 2.5^*^	10.6 ± 2.3
Volume (h/week)	0.2 ± 0.5^*^	10.7 ± 3.6^*^	12.1 ± 3.4
Intensity (average RPE/week)	5.0 ± 0.5^*^	4.9 ± 0.6^*^	5.9 ± 0.6
**STRENGTH**			
Load (AU/week)	1473.3 ± 565.3	1233.3 ± 445.3	1168.9 ± 474.7
Frequency (sessions/week)	2.8 ± 0.9	2.7 ± 0.8	2.6 ± 0.8
Volume (h/week)	4.0 ± 1.3	3.4 ± 1.1	3.2 ± 1.2
Intensity (average RPE/week)	6.2 ± 1.2	6.1 ± 1.2	6.1 ± 1.3
**OTHER**			
Load (AU/week)	2967.2 ± 1606.3^*^	159.8 ± 301.4	0.0 ± 0.0
Frequency (sessions/week)	5.5 ± 1.6^*^	0.7 ± 1.3	0.0 ± 0.0
Volume (h/week)	9.3 ± 3.9^*^	0.7 ± 1.5	0.0 ± 0.0
Intensity (average RPE/week)	5.1 ± 1.4	4.1 ± 2.1	NA

* *p < 0.050 compared to block 3*.

### Time-Trial Performance

**Figure 2:**
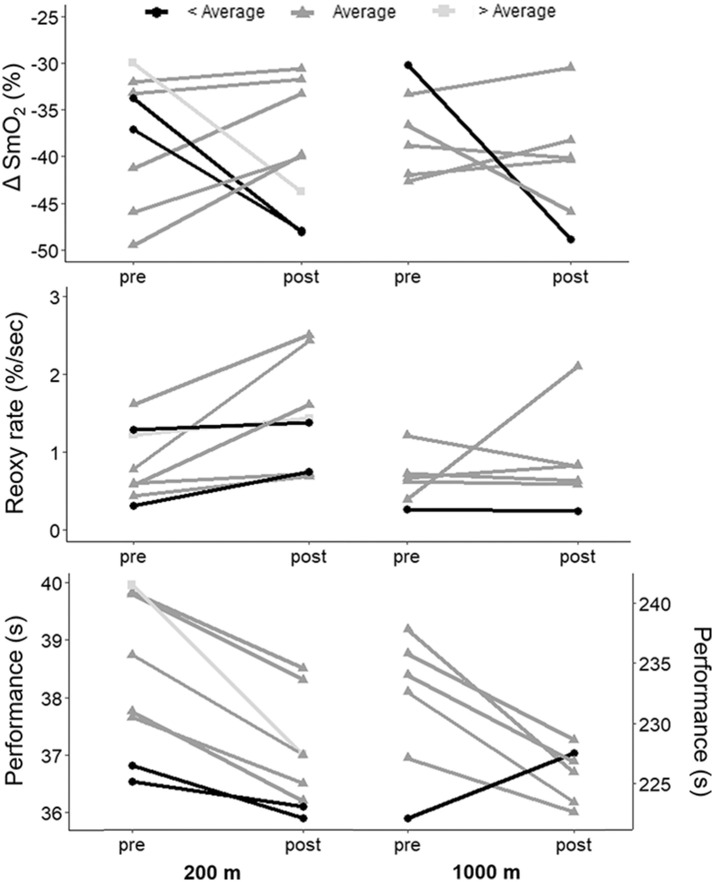
Individual changes in performance, ΔSmO_2_ and reoxy rate in the 200-m and 1,000-m TT with the 3-weeks training camp. Subjects were grouped according to their change in performance, compared to the rest of the group. < Average: subjects whose performance improved by less than (group mean − 0.5*group standard deviation); Average: subjects whose performance improved between (group mean − 0.5*group standard deviation) and (group mean + 0.5*group standard deviation); > Average: subjects whose performance improved by more than (group mean + 0.5*group standard deviation).

Performance parameters from the 200-m and 1,000-m TT are provided in Table 2. Wind and water temperature were similar from pre- to post-training TT (200-m TT: pre 3.5 m/s, from southeast and post 3.4 m/s, from east-southeast; 1,000-m TT: pre 2.5 m/s from southeast and post 3.4 m/s from southeast). In the 200-m TT, performance was improved in all 8 athletes following the training camp (−1.4 ± 0.7 s on average [range −3.0 to −0.4 s], or −3.7 ± 1.7% [range −7.4 to −1.2%], *p* < 0.001, ES: −1.2 [−2.09, −0.31]). In the 1,000-m TT, performance was improved in 6 of the 7 athletes following the training camp (−6.7 ± 5.6 s on average [range −12.3 to 5.4 s], or −2.8 ± 2.4% [range −5.1. to 2.4%], *p* = 0.03, ES: −1.35 [−2.32, −0.38]). These changes in performance are 12.3 times and 9.3 times greater than the smallest worthwhile change for 200-m and 1,000-m, respectively, and 2.6 and 2.0 times greater than the average difference between a 1st place and a 4th place finish at the last Olympic Games. Figure 2 shows individual changes in performance. In both races, athletes who had the best performance before training were the ones who improved the less their performance. In the 200-m TT, the athlete with the worse performance improved the most with training.

**Table 2:** Performance and physiological parameters recorded during TT before and after the training camp.

	**200-m TT**		**1,000-m TT**	
	**Pre**	**Post**	**Pre**	**Post**
Performance	38.4 ± 1.4	36.9 ± 1.0^*^	3:53.0 ± 0:06.6	3:46.3 ± 0:02.5^*^
Baseline SmO_2_ (%max)	76.9 ± 2.9	77.3 ± 2.7	75.6 ± 3.9	74.8 ± 5.0
SmO_2_min (%max)	36.9 ± 7.1	34.7 ± 6.2	36.4 ± 5.1	34.0 ± 4.6
ΔSmO_2_ (%)	−37.8 ± 6.6	−39.4 ± 6.5	−37.2 ± 4.9	−40.0 ± 6.3
Δ[THb] (AU)	0.96 ± 0.34	0.90 ± 0.32	1.33 ± 0.86	1.23 ± 0.43
Deoxy rate (%/sec)	−2.8 ± 1.4	−3.2 ± 0.8	−2.5 ± 0.8	−2.8 ± 0.5
Phase II slope (%/sec)	−0.5 ± 0.5	−0.6 ± 0.2	0.0 ± 0.0	0.0 ± 0.0
Reoxy rate (%/sec)	0.9 ± 0.5	1.4 ± 0.7^*^	0.9 ± 0.5	0.9 ± 0.7
$\dot{\text{V}}$O_2_peak (L/min)	4.17 ± 0.48	4.43 ± 0.54^*^	5.26 ± 0.43	5.45 ± 0.34
Blood [Lactate]				
−1 min	6.4 ± 2.5	5.5 ± 1.4	9.4 ± 3	7.7 ± 2.7
−3 min	10.2 ± 2.1	10.4 ± 2.2	11.8 ± 1.8	10.5 ± 2.8
−5 min	12.9 ± 1.3	11.5 ± 2.9		

* *p < 0.05 between pre- and post-training, AU, arbitrary units*.

### Muscle Oxygenation

**Figure 3:**
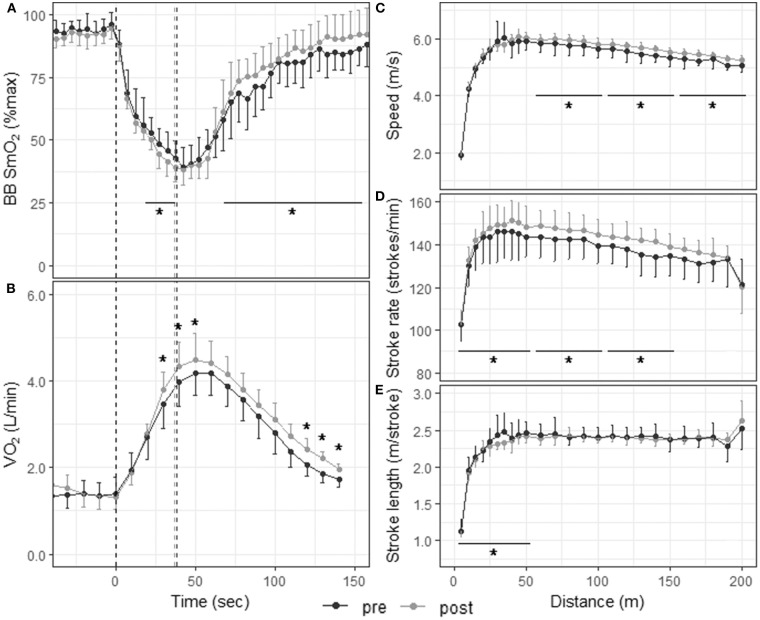
Physiological responses and stroke parameters during the 200-m TT before and after the 3-weeks training camp. First vertical dashed line represents the start of the TT; second dashed line represents the end of the TT post-training and third dashed line the end of the TT pre-training. *p < 0.05 between pre- and post-training TTs. **(A)** Muscle oxygenation in the biceps brachii for 5-s segments 30 s before, during, and 120 s after the 200-m TT; **(B)** O_2_ consumption for 10-s segments 30 s before, during, and 120 s after the 200-m TT; speed **(C)**, stroke rate **(D)**, and stroke length **(E)** for 5-m segments up to 50 m and 10-m segments from 50 to 200 m.

Skinfold thickness at the site of NIRS measurement was 3.3 ± 0.4 mm [range 2.8–4 mm]. Figure 3A displays muscle oxygenation during the 200-m TT before and after the 3-weeks training camp. Baseline SmO_2_ and SmO_2_ in the first half (0–19 s) of the TT did not change with training. However, SmO_2_ in the second half of the TT (19–38 s) was lower after training (43.7 ± 6.5% vs. 47.3 ± 7.4%, *p* = 0.046, ES: −0.51 [−1.35, 0.33]), and SmO_2_ during recovery (from 30 to 120 s after the end of the TT) increased following the camp (84.0 ± 11.1% vs. 79.2 ± 11.5%, *p* < 0.001, ES: 0.45 [−0.39, 1.28]). There was no change in deoxy rate, phase II slope or SmO_2_min with training, but reoxy rate also increased significantly following the training camp (Table 2).

**Figure 4:**
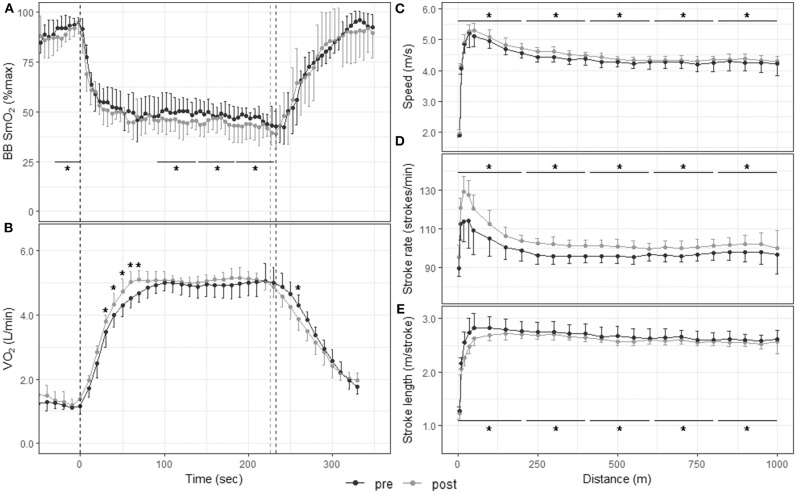
Physiological responses and stroke parameters during the 1,000-m TT before and after the 3-weeks training camp. First vertical dashed line represents the start of the TT; second dashed line represents the end of the TT post training and third dashed line the end of the TT pre-training. *p < 0.05 between pre and post training. **(A)** Muscle oxygenation in the biceps brachii for 5-s segments 30 s before, during, and 120 s after the 1,000-m TT; **(B)** O_2_ consumption for 10-s segments 30 s before, during, and 120 s after the 1,000-m TT; speed **(C)**, stroke rate **(D)**, and stroke length **(E)** for 10-m segments up to 50 m and 50-m segments from 50 to 1,000 m.

Figure 4A displays muscle oxygenation during the 1,000-m TT before and after the 3-weeks training camp. The 1,000-m TT was divided into five segments of same duration. SmO_2_ was similar between pre- and post-training in the first two segments (59.1 ± 13.8% vs. 62.2 ± 14.7%, *p* = 0.233, and 46.8 ± 6.1% vs. 48.6 ± 9.1%, *p* = 0.218). In the last three segments, however, SmO_2_ was significantly lower post-training compared to pre-training (46.0 ± 6.3% vs. 49.3 ± 8.2%, *p* = 0.011, ES: −0.63 [−1.56, 0.31]; 45.1 ± 56% vs. 48.8 ± 5.8%, *p* < 0.001, ES: −0.57 [−1.50, 0.36]; 42.6 ± 7.0% vs. 46.2 ± 5.3%, *p* = 0.001, ES: −0.60 [−1.54, 0.33]). There was no change in deoxy rate, phase II slope, reoxy rate or SmO_2_min with training (Table 2).

Figure 2 shows individual changes in ΔSmO_2_ and reoxy rate with training in the 200-m and 1,000-m TT ranked according to performance change compared to the rest of the group. There was a wide variability in muscle oxygenation response to training among the athletes. In the 200-m TT, athletes whose performance improved similarly to group average had a moderate decrease in ΔSmO_2_ (lower deoxygenation), while athletes whose performance improved more or less compared to group average had a completely different response, where ΔSmO_2_ increased (greater deoxygenation) dramatically with training. There was a very large negative association between pre-training ΔSmO_2_ and change in ΔSmO_2_ with training (*R* = −0.71, *p* = 0.048), the athletes with the lowest ΔSmO_2_ pre-training increasing their ΔSmO_2_ the most with training. There was no association between ΔSmO_2_ and performance in the 200-m TT before training (*R* = 0.33, *p* = 0.43), but a very large association between ΔSmO_2_ and performance post-training (*R* = 0.74, *p* = 0.037). All athletes improved reoxy rate following training, irrespective of the degree of performance improvement. Change in reoxy rate was also associated with pre-training reoxy rate (*R* = −0.74, *p* = 0.035), and there was a small, but not statistically significant association between change in reoxy rate and change in performance (*R* = 0.56, *p* = 0.15).

In the 1,000-m TT, most athletes had small changes in ΔSmO_2_ with training, while the one athlete who did not improve performance had a major increase in ΔSmO_2_ with training. The association between pre-training ΔSmO_2_ and change in ΔSmO_2_ with training did not reach statistical significance in the 1,000-m TT (R = −0.72, *p* = 0.11). There was a small but not statistically significant association between ΔSmO_2_ and performance, both pre- (*R* = −0.51, *p* = 0.30) and post-training (*R* = −0.66, *p* = 0.15). Reoxy rate response to training was variable in the 1,000-m TT. There was no statistically significant association between pre-training reoxy rate and change in reoxy rate with training (*R* = −0.57, *p* = 0.24) and no association between change in reoxy rate and change in performance with training (*R* = 0.43, *p* = 0.40).

### $\dot{\text{V}}$O_2_ and Blood Lactate Concentration

Figures 3B, 4B display the $\dot{\text{V}}$O_2_ response to the 200-m and 1,000-m TT, respectively, before and after training. In the 200-m TT, after the training camp, $\dot{\text{V}}$O_2_ was higher at 30, 40, and 50 s, and at 120, 130, and 140 s during recovery compared to pre-training. $\dot{\text{V}}$O_2_peak was increased by 6.3 ± 6.2% post-training compared to pre-training (*p* = 0.027, ES: 0.51 [−0.32, 1.35]). Blood lactate concentration did not change with training at any time point after the 200-m TT. However, there was a nearly perfect correlation between blood lactate concentration at 3-min post TT and 200-m performance post-training (*R* = −0.92, *p* = 0.0012). In the 1,000-m TT, $\dot{\text{V}}$O_2_ was higher at 30, 40, 50, 60, and 70 s during the effort, and lower at 260 s during recovery. There was only a tendency for a higher $\dot{\text{V}}$O_2_peak after training (4.4 ± 5.8%, *p* = 0.141, ES: 0.48 [−0.41, 1.37]). There was also a trend for a lower blood lactate concentration 1 min after the 1,000-m TT post-training (*p* = 0.066, ES: −0.59 [−1.49, 0.31]), and a very large association between training associated change in blood lactate concentration at 3-min post TT and change in 1,000-m completion time (*R* = 0.79, *p* = 0.034).

### Stroke Parameters

Figures 3C–E, 4C–E depict speed, stroke rate and stroke length, respectively, during the 200-m and 1,000-m TT before and after training. The 200-m TT was divided into four 50-m segments. Speed was not different after training in the first quarter of the TT (5.19 ± 1.24 m/s vs. 5.17 ± 1.28 m/s, *p* = 0.597, ES: 0.02 [−0.81, 0.84]], but was higher in the last three quarters of the TT (5.93 ± 0.20 vs. 5.77 ± 0.33 m/s, ES: 0.58 [−0.26, 1.42]; 5.68 ± 0.18 vs. 5.48 ± 0.32 m/s, ES: 0.79 [−0.07, 1.64]; 5.38 ± 0.15 vs. 5.20 ± 0.29 m/s, ES: 0.80 [−0.05, 1.66] all *p* < 0.001). Stroke rate was higher in the first 3 quarters of the race (142 ± 16 vs. 139 ± 17 strokes/min, ES: 0.2 [−0.63, 1.02]; 147 ± 7 vs. 142 ± 9 strokes/min, ES: 0.59 [−0.25, 1.43]; 142 ± 6 vs. 136 ± 9 strokes/min, ES: 0.73 [−0.12, 1.58], all *p* < 0.001), and tended to be higher in the last quarter (133 ± 10 vs. 130 ± 11 strokes/min *p* = 0.076, ES: 0.25 [−0.58, 1.07]). Stroke length was lower in the first quarter of the race (2.15 ± 0.39 vs. 2.20 ± 0.44 m/stroke, *p* = 0.012, ES: −0.12 [−0.94, 0.71]), but did not change with training in the last 3 quarters (all *p* > 0.250). The 1,000-m TT was divided into five 200-m segments. After training, speed and stroke rate were higher and stroke length lower for all segments compared to before training (all *p* < 0.001).

### Association Between Training Load and Changes in Performance, Physiological, and Stroke Parameters

**Figure 5:**
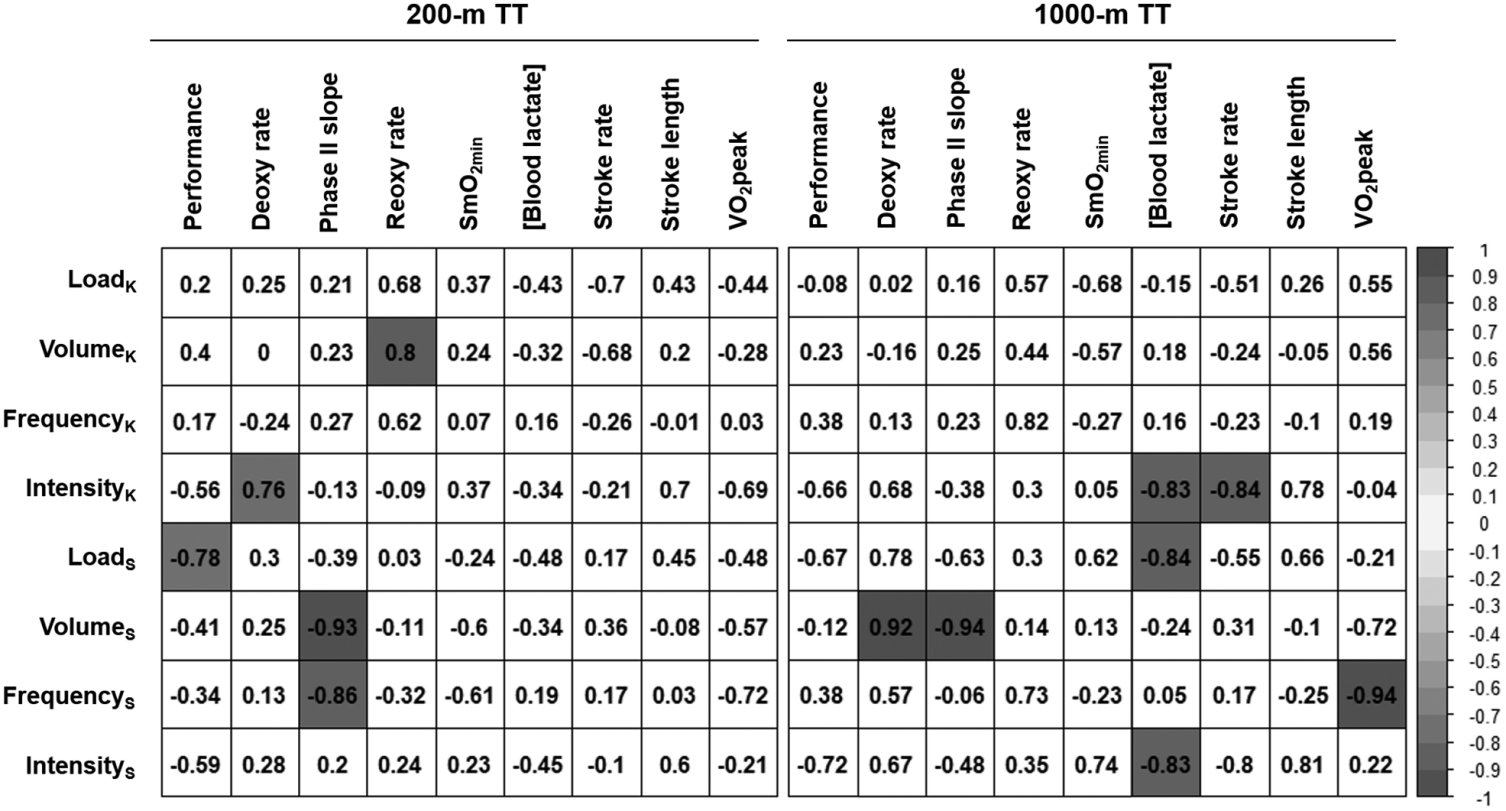
Association between training load during the 3-weeks training camp and changes in performance, physiological and stroke parameters during the 200-m and 1,000-m TT between pre- and post-training time trials. Load_K_: Kayak training load (AU/week), Volume_K_: Kayak training volume (h/week), Frequency_K_: Frequency of kayak sessions (sessions/week), Intensity_K_: average intensity (average RPE) of kayak sessions, Load_S_: Strength training load (AU/week), Volume_S_: Strength training volume (h/week), Frequency_S_: Frequency of strength training sessions (sessions/week), Intensity_S_: average intensity (average RPE) of strength training sessions, [Blood lactate]: blood lactate concentration at 3-min post-race. Values are Pearson's correlation coefficients, and colored values represent *p* < 0.05.

Pearson's correlation coefficients were calculated to assess the associations between the training load performed during the 3-weeks training camp and the changes in physiological and stroke parameters from pre- to post-training TTs (Figure 5). For the 200-m TT, a higher strength training load during the training camp was associated with a larger increase in performance (*p* = 0.038). A greater volume and frequency of strength training were also associated with a steeper phase II slope (*p* = 0.002 and *p* = 0.013, respectively). A higher intensity (higher average RPE) in kayak sessions was associated with a slower deoxy rate (*p* = 0.047), while a greater volume of kayak training was associated with a faster reoxy rate (*p* = 0.033). Finally, a higher kayak training load and volume were associated with a smaller increase in stroke rate, but these associations did not reach statistical significance (*p* = 0.082 and *p* = 0.093, respectively).

In the 1,000-m TT, there was a moderate association between average training intensity in both kayak and strength training and TT performance, but this association was not statistically significant (*p* = 0.157 and *p* = 0.108, respectively). A higher training intensity in both kayak and strength sessions was also associated with a lower blood [lactate] after the race (*p* = 0.040 and *p* = 0.041, respectively), and a high intensity in kayak session was also associated with a smaller increase in stroke rate (*p* = 0.036). A higher strength training frequency was strongly associated with a decrease in $\dot{\text{V}}$O_2_peak (*p* = 0.005). Finally, a higher strength training volume was associated with a slower deoxy rate (*p* = 0.029) and an acceleration of phase II slope (*p* = 0.016).

## Discussion

In elite sprint kayakers, a 3-weeks winter training camp imbedded within the annual periodization with a ~20% increase in training load and intensity resulted in a ~4% improvement in 200-m TT and a ~3% improvement in 1,000-m TT. Performance improvement in the 200-m was concurrent with a reduced SmO_2_ and increased $\dot{\text{V}}$O_2_ at the end of the TT, an increase in $\dot{\text{V}}$O_2_peak and an increase in post-TT reoxygenation rate. The 1,000-m TT performance improvement was concurrent with a reduced SmO_2_ in the last half of the TT and an increased $\dot{\text{V}}$O_2_ in the first minute of the TT.

### Training-Induced Changes in Physiological Parameters During the Time Trials

To our knowledge, this study is the first to assess changes in muscle oxygenation with training in elite kayakers. We observed a 3% improvement in 1,000-m performance, concurrent with a reduction in *biceps brachii* SmO_2_ in the last portion of the race. This is coherent with two studies on trained cyclists, where the increase in 20-km TT performance following a 7-days taper (Neary et al., 2005) or a 3-weeks high-intensity endurance training program (Neary et al., 2002) was associated with a reduction in *vastus lateralis* oxyhemoglobin ([HbO_2_]). However, in mountain bikers undergoing a 4-week HIIT program, authors stated (data is not shown) that there was no change in *vastus lateralis* peak deoxyhemoglobin ([HHb]) during an incremental test, despite an 8% improvement in maximal aerobic power (Gendron et al., 2016). This difference may come from the nature of the aerobic test (TT vs. incremental test).

The improvement in 200-m TT performance and reduction in *biceps brachii* SmO_2_ with training is coherent with other studies evaluating the effect of training on muscle oxygenation during “anaerobic” performance. In 10 elite women field hockey players, 6 weeks of cycling HIIT were associated with an increased deoxygenation (larger decrease in tissue saturation index, ΔTSI), and an increased O_2_ extraction (larger increase in [HHb], Δ[HHb]) during repeated 30-s all-out sprints (Jones et al., 2015). Similarly, there was an increased ΔTSI and increased Δ[HHb] during repeated 10-s cycling sprints between pre- and mid-season in trained rugby players (Jones and Cooper, 2014), and an increased ΔTSI during 15-s shuttle runs following a 6-weeks HIIT or small sided games intervention in 20 junior male basketball players (Delextrat et al., 2018). Taken together, our current results indicate that elite kayakers display similar adaptations to a short-term intensified training period as varied athletic populations.

Even though ΔSmO_2_ did not change on average with training, there was a very large association between ΔSmO_2_ and 200-m TT performance after training, a greater ΔSmO_2_ being associated with higher performance. This is consistent with previous studies where Δ[HHb] in the latissimus dorsi muscle was related with performance in trained kayakers (Borges et al., 2015; Paquette et al., 2018). However, we did not find such an association in the 1,000-m TT, in contrast to previous studies (Borges et al., 2015; Paquette et al., 2018). Since this study is the first to assess elite senior kayakers, this difference may come from athletes in this study being specialists of shorter or longer distances, contrary to junior athletes or provincial level athletes, training and racing all distances. In such an elite population, ΔSmO_2_ seems to be a better predictor, and so a better training aid when training for 200-m rather than 1,000-m races. Since SmO_2_ represents the balance between O_2_ delivery and extraction at the muscle level (Ferrari et al., 2011), a decrease in SmO_2_ may originate from both reduced delivery and/or increased extraction. However, the most plausible explanation for the decreased SmO_2_ in the 200-m and 1,000-m TT is an increase in O_2_ consumption by the locomotor muscle, due to training-induced increase in mitochondrial content (Granata et al., 2018), resulting in improved O_2_ extraction. This is supported by studies where increased ΔTSI was associated with an increase in extraction (Δ[HHb]) (Jones and Cooper, 2014; Jones et al., 2015), and no change (Jones et al., 2015) or an increase (Faiss et al., 2015) in [THb], indicating that O_2_ delivery is unlikely to decrease with training (especially when performance is enhanced).

In the 200-m TT, increased $\dot{\text{V}}$O_2_ and decreased SmO_2_ both occurred in the last portion (last 10–15 s) of the TT. Even though we did not measure cardiac output in this study to assess the role of central adaptations, our data suggests that increased $\dot{\text{V}}$O_2_ during the 200-m TT is explained at least partly by an increase in O_2_ extraction by the muscle, and therefore, by peripheral adaptations. In the 1,000-m TT, $\dot{\text{V}}$O_2_ increased early in the effort (within the first minute), while SmO_2_ declined only in the last 3/5 of the TT. The increase in $\dot{\text{V}}$O_2_ without a change in SmO_2_ in the first portion of the TT suggests an enhanced O_2_ delivery at exercise onset, potentially due to the higher speed and stroke rate recorded at the beginning of the effort post-training (see Figures 4C,D). In the last portion of the race, the lower SmO_2_ despite unchanged systemic $\dot{\text{V}}$O_2_ may indicate a decreased O_2_ delivery to the muscle near the end of the TT, which could be compensated by a greater extraction. A similar interpretation was proposed by another research group, where an increase in Δ[HHb] to Δ$\dot{\text{V}}$O_2_ ratio was interpreted as an “impairment in O_2_ delivery relative to O_2_ utilization” (Murias et al., 2010). The reduced O_2_ delivery to the biceps may be the effect of an increase in systemic O_2_ utilization (through ventilation and cardiac work, other locomotor skeletal muscles not recorded in this study, thermoregulation, gluconeogenesis, etc.), competing with the active muscles for O_2_ delivery (Dempsey et al., 2006). There was no change in Δ[THb] with training in the 1,000-m TT, so no evidence of reduced O_2_ delivery to the muscle, but [THb] is not a direct measure of flow. Also, while recent studies have found that the Moxy monitor provides credible and reliable SmO_2_ values (McManus et al., 2018), Moxy derived [THb] values barely change with exercise and therefore are probably not a valid indicator of blood volume changes during exercise (Crum et al., 2017). Thus, the most likely explanation for the progressive tissue desaturation following training in both TTs is an increase in muscle oxygen consumption due to an increase in mitochondrial content and/or function (Jacobs et al., 2013). This is consistent with other authors suggestion that peripheral, rather than cardiac, adaptations are likely responsible for the high-intensity training-induced improvement in performance in well-trained endurance athletes (Laursen and Jenkins, 2002).

In the present study, training related changes in ΔSmO_2_ were highly variable between athletes. Some (Jones and Cooper, 2014), but not all (Jones et al., 2015) studies performed in elite athletes report such a variability in muscle oxygenation response to training. In the present study, some of the inter individual variation was explained by pre-training ΔSmO_2_, suggesting physiological adaptations to training may differ depending on baseline characteristics of the athlete, and we may speculate that some athletes in this study had already maximized ΔSmO_2_.

### Training Associated Changes in Physiological Parameters at Exercise Onset and Cessation

Rate of deoxygenation in the first 12-s of both 1,000-m and 200-m TT was ~3%/s, which is comparable to values reported during six 30-s cycling sprints, ranging from ~2.5 to ~5% (Buchheit et al., 2012). While rate of deoxygenation has been associated with citrate synthase activity (Puente-Maestu et al., 2003), and has been shown to increase with training in recreationally-active adults (Bailey et al., 2009), it did not change with training in the current study. However, we reported an increased $\dot{\text{V}}$O_2_ from 30 to 50 s in the 200-m TT and from 30 to 70 s in the 1,000-m TT, indicative of an acceleration of $\dot{\text{V}}$O_2_ kinetics after training. This may be explained by the faster phosphagen depletion necessary to achieve the higher speed, and is a well-described effect of endurance training (Phillips et al., 1995). We speculate that the discrepancy between current NIRS-derived data and $\dot{\text{V}}$O_2_ data could come from the Moxy-derived SmO_2_ variable which represents a balance of O_2_ delivery to extraction vs. the alternative [HHb] that some authors suggest better reflects deoxygenation within the muscle (DeLorey et al., 2003).

In the current study, reoxygenation rate in the 30 s following the completion of the 200-m TT was faster after training. This finding is also consistent with other studies where training was associated with faster reoxygenation after repeated sprints, whether 2 × 15-s sprint with 15-s recovery (Buchheit and Ufland, 2011; Delextrat et al., 2018) or 5 × 30-s sprint with 4-min recovery (Jones et al., 2015). A faster increase in SmO_2_ post-effort suggests that the O_2_ supply/O_2_ consumption balance is being restored more rapidly, and it has been suggested that the main factors accounting for such change are improved muscle oxidative capacity (Puente-Maestu et al., 2003), muscle blood flow and capillarization (Kime et al., 2003). These adaptations of the peripheral system may very well have taken place during the kayak-specific 3-weeks training camp.

### Associations Between Training Load and Training Adaptations

An interesting finding of the current study is a positive association between strength-training load during the training camp and 200-m TT performance improvement. These results confirm the importance of strength training for relatively short events, and are in line with various studies were 200-m performance was strongly associated with upper-body strength (van Someren and Palmer, 2003; Pickett et al., 2018) or dimensions (van Someren and Palmer, 2003; van Someren and Howatson, 2008). Conversely, there was a strong negative correlation between strength training frequency and change in $\dot{\text{V}}$O_2_peak during the 1,000-m TT, with a frequency of ~3 sessions/week being associated with reduced $\dot{\text{V}}$O_2_peak during 1,000-m TT. It has been suggested that resistance training-induced fatigue may impair the quality of endurance training sessions during concurrent training, thereby limiting optimization of endurance development (Doma et al., 2017), especially during a training camp where the kayak specific load was dramatically increased.

A higher volume of kayak training was also associated with an acceleration of reoxy rate after the 200-m TT, which is in line with the results from a study where an increase number of bike training sessions was correlated with an improvement in post-exercise recovery of oxygenation in rugby players (Jones and Cooper, 2014). These authors also reported that players who exercised at the highest power tended to decrease their muscle oxygenation to a greater extent (Jones and Cooper, 2014), but there was no clear association between training intensity and oxygenation in the current study.

### Limits

While acknowledging that the study was performed during an actual training camp which offers the opportunity to assess real-world changes in elite athlete performances and provides very practical insights to coaches and athletes, results must be interpreted with the following limits. Our choice to use elite athletes limited the number of participants, and therefore, the small sample size may not have provided sufficient statistical power to detect some of the subtle training-induced physiological changes associated with the 3–4% improvement in on-water TT performance. Even though the chosen statistical approach is well-suited for the analysis of low sample size data in reference to practical benchmarks (Hopkins et al., 2009), the presented correlations must be considered with care. The lack of a control group is another limitation of the present study, but the training-induced changes reported here are significant and larger than variation coefficients reported in elite sprint kayak athletes (Bonetti and Hopkins, 2010), therefore we are confident they represent true changes. It is also extremely difficult to select a control group of similar kayak level which remains “at home” for comparison purpose due to training calendar considerations and national team selection obligations. Moreover, the goal was to assess changes in physiological and performance following a habitual training camp, but future studies should compare the effect of different training blocks on these parameters. Another limitation of this study is the utilization of a single muscle for muscle oxygenation assessment. In a previous study, we showed that combining oxygenation data from *biceps brachii, vastus lateralis*, and *latissimus dorsi* provided stronger correlations with kayak performance, compared to investigating a single muscle (Paquette et al., 2018). While the results of our study are limited by the use of only one muscle, they better reflect the monitoring that can be performed with elite athletes in the daily training environment, reducing the number of devices a team needs to buy and reducing installation time before every test. While upper-body muscles primarily involved in the kayak stroke are the *latissimus dorsi, triceps brachii* and the anterior deltoids (Fleming et al., 2012), *biceps brachii* also contributes to the force production, as suggested by the important muscle deoxygenation that occurs during kayaking in this muscle (Paquette et al., 2018). The *biceps brachii* is an easier muscle than the *latissimus dorsi* to assess with NIRS, since the monitor placement is easier and signal quality more optimal. Finally, while the use of session RPE for training load quantification has been validated against more objective training load measurements (van Erp et al., 2019), and was used in this study to allow for comparison of training load from different training modalities, it should be acknowledged that RPE is not a direct measure of training intensity, as it is also influenced by training volume.

## Conclusion

A 3-weeks kayak-specific training camp integrated in the yearly training plan induced clear increases in 200-m and 1,000-m on-water TT performance, reduced SmO_2_ and increased $\dot{\text{V}}$O_2_. The non-invasive NIRS measurements strongly suggest that peripheral adaptations occurred in these athletes with the proposed training plan. Our results further support the use of portable NIRS as a monitoring tool to track training effects on muscle oxygen extraction in elite athletes.

## Data Availability Statement

The datasets generated for this study are available on request to the corresponding author.

## Ethics Statement

The studies involving human participants were reviewed and approved by Comités d'éthique de la recherche avec des êtres humains de l'Université Laval. The patients/participants provided their written informed consent to participate in this study.

## Author Contributions

All authors contributed to the study conception and design. Material preparation, data collection, and analysis were performed by MP. The first draft of the manuscript was written by MP and all authors commented on previous versions of the manuscript. All authors read and approved the final manuscript.

## Conflict of Interest

The authors declare that the research was conducted in the absence of any commercial or financial relationships that could be construed as a potential conflict of interest.
